# Faculty Beliefs about the Goals of Doctoral Education in Chemistry: Results from a National Survey

**DOI:** 10.1021/acs.jchemed.6c01087

**Published:** 2026-10-13

**Authors:** Pallavi Nayyar, Benedicta Donkor, Jordan Harshman

**Affiliations:** † Department of Chemistry, University of Iowa, Iowa City, Iowa 52246, United States; ‡ Department of Chemistry, Middle Tennessee State University, Murfreesboro, Tennessee 37132, United States

**Keywords:** Chemical Education Research, Graduate Education, Reform, Faculty

## Abstract

Doctoral education in chemistry (DEC) underpins the United States’ research capacity, innovation ecosystem, and preparation of a highly skilled scientific workforce. The goals that guide doctoral education carry significant national implications as they determine which careers in academia, industry, government, and science-adjacent sectors future scientists are well-prepared for and pursue. In this study, we report results from a national survey of research-active chemistry faculty in the United States examining beliefs about the goals of DEC and their relationship to personal and contextual factors. Faculty demonstrated strong consensus around student-centered goals, including developing independent scientists and preparing students for varying career pathways. Comparatively, endorsement of the institution-focused goal of enhancing rank or reputation was more variable and less strongly prioritized but still a present goal of doctoral programs. No evidence for differences based on academic rank and graduate-education related service role was found. Together, these findings provide national-level evidence of faculty alignment around goals of DEC and offer insight into how consensus and variability in faculty beliefs inform opportunities for doctoral education reform. This pattern highlights opportunities to focus reform efforts on aligning doctoral program structures with faculty-endorsed, student-centered goals.

## Graphical abstract

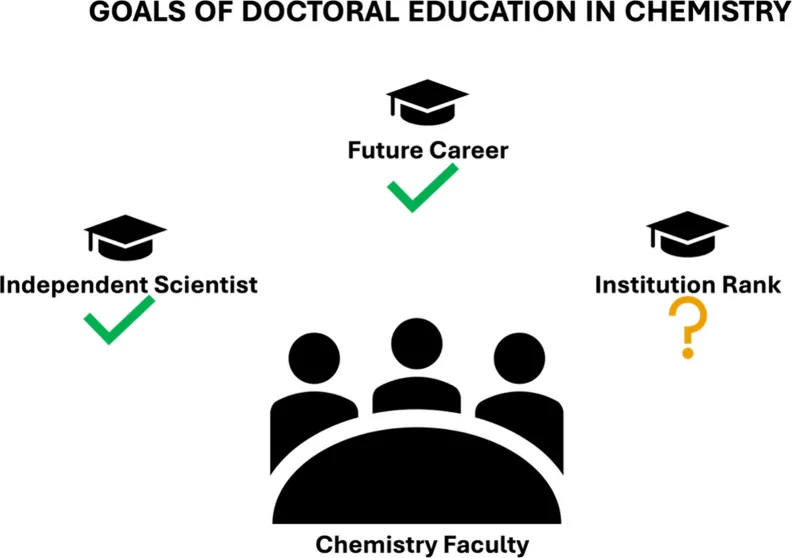

## Introduction

Doctoral education in chemistry (DEC) underpins the research capacity and innovation ecosystem of the chemical sciences, producing scientists who advance fundamental discovery, develop new technologies, and contribute across academia, industry, government, and emerging professional sectors. Historically, chemistry doctoral programs have been organized around a stable set of programmatic elements: original research, advanced coursework, qualifying or candidacy examinations, seminars, and teaching assistantships. These collectively socialize students to the norms and practices of this discipline.^1–5^ Despite this structural consistency, the goals of doctoral education have come under increasing scrutiny as the career outcomes of chemistry Ph.D. graduates have spread to many sectors and workforce demands have evolved.^6–8^

At a national level, the Graduate STEM Education for the 21st Century report from the National Academies of Sciences, Engineering, and Medicine (NASEM) emphasizes that doctoral programs must prepare graduates not only for independent research but also for a broad range of professional roles and societal contributions.^6^ This report calls for clearer articulation of doctoral goals, explicit alignment between program structures and intended outcomes, and greater accountability for how doctoral educationprepares graduates for contemporary careers. Within chemistry specifically, the American Chemical Society (ACS) has echoed these concerns through presidential commission reports and graduate education resources, articulating doctoral education goals that include deep disciplinary expertise, critical thinking, ethical and safe research practice, effective communication, mentoring, and career readiness across sectors.^9–11^ Therefore, it is theorized that the primary goals of doctoral programs in STEM (NASEM) and Chemistry (ACS) are to engender independent scientists and prepare students well for future careers. Nevertheless, bridging policy-level visions and program-level realities requires participation from multiple stakeholders, including doctoral students, faculty, institutions, funding agencies, professional societies, and the broader scientific workforce.

Faculty occupy a particularly vital position in determining the roles of doctoral programs as they mentor doctoral students, evaluate progress, interpret external expectations within local contexts, and ultimately have a great deal of autonomy regarding the design of their programs.^12–19^ While prior national reports theorize the two primary goals, prior research^20^ has verified that faculty not only express these two goals but also define the goals of doctoral programs as obtaining certain sets of skills (e.g., critical thinking, gaining expertise, etc.) and improving the reputation and rank of their chemistry departments. To date, we do not know the extent to which faculty believe what priority or weight each goal should be given. In this study, we examine research-active chemistry faculty’s beliefs about the relative importance of these goals using data from a national survey. This work provides empirical evidence regarding where faculty consensus and variation exist in conceptions of goals of DEC. These beliefs are incredibly important as they are primary drivers of the practices that individual faculty and departments incorporate into doctoral education.^21^

## Theoretical Framework

The current study is situated within the teacher-centered systemic reform (TCSR) model of educational change.^21^ The TCSR model conceptualizes educational practice and change as driven primarily by instructor thinking, defined as their beliefs, values, and knowledge about teaching and learning. In TCSR, faculty thinking mediates the influence of personal factors such as career stage and professional experience and contextual factors such as departmental norms, institutional incentives, and governance roles on the enacted practices. In the context of DEC (see Figure 1), TCSR provides a lens for examining how faculty conceptualize the goals of doctoral education (thinking) and how these beliefs interact with academic rank (personal) and service role (contextual) factors. While there are certainly other contextual and personal factors that likely drive beliefs and practices, we limit the scope of our study to focus on those that are most readily and reliably measured.

**Figure 1:**
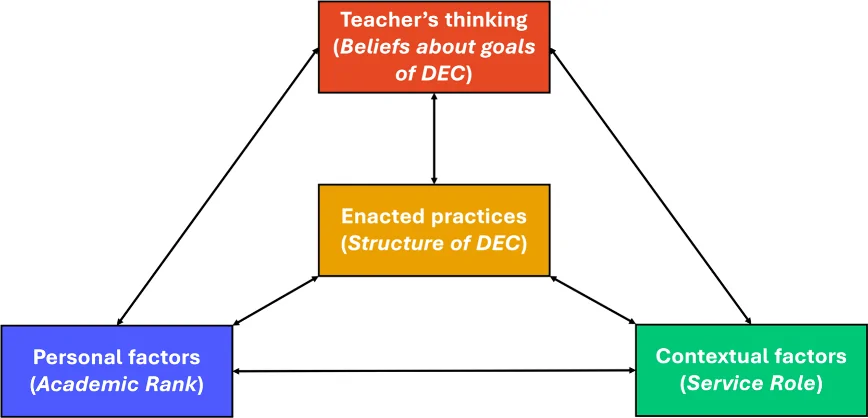
TCSR Model within the DEC context as applicable to the current study.

## Hypothesis and Research Questions

Given the prior literature, we expect faculty beliefs to be distributed across three goals of DEC: the development of independent scientists (goal IS), the preparation of students for their future careers (goal FC), and the improvement of the rank or reputation of their institution (goal IR). Below, we provide a background supporting each as a goal of DEC and our hypothesis about how faculty may prioritize each. Following the TCSR model (Figure 1), the first three hypotheses examine faculty beliefs (i.e., thinking) regarding the relative importance of Goals IS, FC, and IR. The final hypothesis considers whether these beliefs vary as a function of personal (academic rank) and contextual factors (graduate-education service role) identified in the TCSR model (Figure 1). Table 1 also provides a snapshot of the relationships among TCSR construct, each goal, and our hypothesis.

**Table 1:** Relationship among TCSR Construct, Each Goal and Hypothesis

*TCSR Construct*	*Hypothesis*	*Relationship*	*Hypothesis in context*
Teacher’s thinking (i.e., beliefs)	Hypothesis IS	Whether faculty thinking about DEC’s goal centers on scientific independence	This hypothesis tests the content of faculty thinking (Figure 1) regarding the historically dominant aim of doctoral education.
	Hypothesis FC	Whether an externally driven concern (workforce/career readiness) has been absorbed into faculty thinking as a core belief, not just an outside pressure	This hypothesis examines whether an originally external expectation has become integrated into faculty thinking itself, per TCSR’s bidirectional model.
Teacher’s thinking, shaped by contextual factors (institutional incentives/prestige structures)	Hypothesis IR	Whether an institution level contextual pressure (rank, reputation) has been internalized into individual faculty belief or remains an external expectation	This hypothesis examines whether institutional pressures (Figure 1) have been internalized into faculty thinking versus remaining external.
Personal factors (academic rank) and contextual factors (graduate-education service role) into teacher’s thinking	Hypothesis Academic rank & Service role	Directly tests the TCSR pathway from personal/contextual factors to faculty thinking	This hypothesis examines whether faculty’s beliefs vary based on personal factor and contextual factor (Figure 1).

### Independent Scientist (Goal IS)

The historical design of the PhD emerged from a liberal arts philosophy emphasizing intellectual development and scholarly inquiry, with the Doctor of Philosophy conceived as a research degree centered on original contribution and intellectual autonomy.^22–25^ Within chemistry, this conception has been operationalized through intensive research training intended to cultivate students’ capacity for independent scholarly work as Burke states “*The goal of the PhD chemistry program is to develop inquisitive, logical chemical scientists who can independently design and carry out carefully planned, original research and then report the significance of the results clearly.”* ^24^ Independence reflects students’ increasing ownership over problem formulation, methodological decisions, and interpretation of results, marking a transition from guided training to self-directed expertise. This framing has remained remarkably consistent across decades of reports related to DEC.^3^

#### Hypothesis IS

Given the historical centrality of scientific independence, we hypothesize that faculty may strongly endorse developing independent scientists as a primary goal of doctoral education while differing in how this goal is prioritized relative to other goals.

### Future Careers (Goal FC)

Preparing doctoral students for future careers represents a student-centered goal, grounded in ethical and economic expectations that PhD graduates should be able to secure gainful employment across a range of professional contexts. This emphasis reflects increasing national concern about workforce preparedness and accountability in graduate education.^6,10^ However, the extent to which an emphasis on developing independent scientists helps or hurts a more vocation-oriented goal is disputed. The Doctor of Philosophy was neither intended nor structured as a professional credential, placing career readiness in tension with the original educational mandate of doctoral programs.^26^ This tension is magnified by labor market conditions: more than 3,350 chemistry PhDs are awarded annually in the United States, while only ∼400–600 permanent academic positions become available each year, alongside uncertain opportunities across industry and government sectors.^7,27^ In practice, doctoral admissions are often guided by the availability of teaching and research assistantships rather than by demonstrable workforce demand, further complicating the alignment between training and employment outcomes.^3^ Consequently, faculty beliefs about career preparation may vary depending on whether this goal is viewed as a core educational responsibility or as an external economic concern. Alternatively, developing independent scientists is often assumed to provide students with transferable skills to most career sectors. If true, focusing solely on developing an independent scientist will naturally make them competitive for future careers and the two goals are symbiotic rather than in competition with one another.

#### Hypothesis FC

We hypothesize that faculty endorsement of preparing students for future careers will vary relative to other goals, reflecting differing views on whether career competitiveness constitutes a primary goal or a secondary outcome beyond its historical design.

### Institutional Rank (Goal IR)

The goal of enhancing institutional rank or reputation can be understood as intertwined with the structure and incentives of doctoral education in chemistry as prestige is associated with tangible benefits for departments, faculty, and research programs. Higher-ranked programs attract more external funding and prominent collaborators, reinforcing their position within the research ecosystem;^2^ thus fulfilling and advancing the research mission of the institution. Empirical evidence suggests that prestige operates as a self-reinforcing mechanism in academia: recent analyses indicate that approximately 80% of tenure-track faculty at research-intensive institutions are trained at just 20% of doctoral programs, highlighting the extent to which prestige shapes academic career mobility and reproduces disciplinary hierarchies.^28^ Similar patterns have been observed across scientific fields, where institutional rank is generated through mechanisms (such as publication volume, grant funding, and faculty accolades) that do not necessarily align with student development.^29^ Importantly, institutional rank is generated through mechanisms (such as publication volume, grant funding, and faculty accolades) that do not necessarily align with student development.^29^ As a result, enhancing reputation may serve departmental and disciplinary interests more directly than the lived educational experiences of doctoral students.

#### Hypothesis IR

Accordingly, we hypothesize that faculty beliefs about institutional rank as a goal of doctoral education will be more variable than beliefs about goals that are more student-centered, reflecting differing views on whether prestige represents a legitimate educational goal or a secondary outcome of DEC.

### Varying of Beliefs by Background

Prior research on faculty careers, professional socialization, and institutional engagement provides a theoretical basis for hypothesizing that both academic rank and participation in doctoral education service roles may meaningfully influence faculty beliefs about the goals of DEC. Graduate school and early career experiences socialize faculty into disciplinary norms surrounding research, mentoring, and scholarly success, shaping initial conceptions of doctoral training and its purposes.^30^ As faculty progress through academic ranks, their professional roles, autonomy, and engagement with institutional priorities often evolve, which may in turn influence how they interpret and prioritize doctoral education goals.^31,32^ Similarly, participation in service roles related to graduate education (e.g., recruiting, admissions, graduate program officers, department heads, etc.) increases faculty exposure to program-level decision-making, accountability structures, and institutional expectations, potentially shaping beliefs about doctoral education through sustained interaction with these contexts.

Within the TCSR framework, academic rank functions as a personal factor reflecting differences in professional experience and career stage, whereas doctoral education service roles function as a contextual factor that conditions how faculty encounter and interpret institutional structures. Together, these factors are expected to shape faculty thinking by influencing how educational goals are understood, negotiated, and enacted within doctoral programs.

#### Hypothesis Academic Rank and Service Role

Accordingly, we hypothesize that faculty beliefs about the goals of doctoral education in chemistry would vary based on academic rank and participation in graduate education service roles.

To test our hypotheses, we investigated the following two research questions:

RQ1. To what extent, and with what degree of consensus, is faculty thinking organized around the three theorized goals of DEC?

RQ2. To what extent do personal factors (academic rank) and contextual factors (graduate education service role) relate to variation in faculty thinking about the goals of DEC?

## Methods

### Survey Development

A survey was developed to elicit faculty beliefs about goals, programmatic elements, and challenges within context of their doctoral program in chemistry. The survey items were based on previous qualitative results from Donkor et al. 2024 ^20^ and Collini et al. 2025.^33^ For the current report, the focus will be limited to items about goals of DEC (listed in Box [Boxed-text box1]). The survey items were designed based on standards for educational and psychological testing^34^ as follows.

1 **Survey items about goals of DEC**

Please state the degree to which you agree or disagree with the following statements: You will have a chance to write in your goal if you feel that none of these reflect the primary goals of your doctoral program.

I believe the primary goal of the doctoral program in my department is to···

- ...educate/train students who are competitive for future careers
- ...educate/train students who are independent scientists
- ...develop students who can improve our institutional rank or reputation

**Response choices:** • Strongly disagree • Somewhat disagree • Somewhat agree • Strongly agree • Not applicable

#### Evidence Based on Test Content

4-point Likert scale survey items were developed based on the results of our group’s previously conducted qualitative study.^20^ Direct quotes were used to inform the wording of the items. For example, the goal “independent scientist” came from quotes observed in that prior study such as “able to independently formulate research questions, strategies for answering research questions, and interpreting experiments” (Participant 1) and “competitive for future careers” from quotes such as “want to train our graduate students to be competitive in the job market” (Participant 2). An additional open-ended item was included to give participants the opportunity to report any DEC goals not represented in the survey items.

#### Evidence Based on Response Process

Evidence based on the response process was gathered through think-aloud interviews with three chemists (two of which were faculty members) who hold roles directly related to graduate education. Participants were asked to articulate their interpretation of each survey item and explain the reasoning underlying their selected responses. Feedback from these discussions indicated that faculty interpreted the items as eliciting their beliefs about what the goals of doctoral education in chemistry should be while simultaneously reflecting on how doctoral education is enacted within their departments (i.e., what the goals are in practice). This blending of beliefs and enacted practices was expected, as the TCSR model posits a bidirectional relationship between faculty beliefs and instructional or programmatic practices with each shaping and reinforcing the other. Overall, the response process evidence suggests that the survey items functioned as intended by capturing faculty beliefs about the goals of doctoral education within the context of their lived departmental experiences.

#### Evidence Based on Relationship to Other Variables

Additional validity evidence was established by examining the conceptual alignment between survey items in the present study and related constructs measured in prior national surveys. Items in our survey parallel competencies assessed in ACS 2019 survey of graduate students.^35^ Although direct quantitative associations (e.g., correlations) are not appropriate due to differences in target populations (research-active faculty versus graduate students), conceptual correspondence provides support for the interpretation of the items. For example, the item assessing the goal of educating or training students to become independent scientists aligns with the ACS competency Develop Scientific and Technological Literacy and Conduct Original Research, which includes items such as “Able to design a research strategy, including relevant quantitative, analytical, or theoretical approaches, to explore components of the problem.” Similarly, the item assessing preparation for future careers aligns with the ACS competency Develop Leadership, Communication, and Professional Competencies, measured through items such as “Developed the ability to work in collaborative and team settings involving colleagues with expertise in other disciplines and from diverse cultural and disciplinary backgrounds.” This alignment suggests that the survey items are consistent with articulated expectations for what doctoral students should gain from their training, supporting their use as indicators of goals of DEC.

### Data Collection

The study was approved as exempt by the University of Iowa’s Institutional Review Board (IRB-02; ID# 202510128). A list of research-active faculty (name, email, rank and area of research) was compiled using departmental webpages from a list of 212 PhD-granting institutions. This resulted in a final database of 4,203 research-active faculty in the United States. Email invites to participate in the survey were sent during Fall 2025 and Spring 2026 via Qualtrics. Faculty provided informed consent to participate in the study. A total of 202 responses were received. Faculty first rated their agreement with the three DEC goals on a 4-point Likert scale (Strongly disagree to Strongly agree). They were then asked to rank the goals by priority. The survey concluded with demographic questions regarding their graduate education service roles and academic rank among other variables. No safety hazards were associated with participation in the study.

### Data Analysis

Missing data were handled using pairwise deletion, meaning a participant’s response was only excluded from a specific analysis if the needed variable was missing; rather than being dropped from the entire data set. This approach preserves as much data as possible but means that the sample size (N) is not identical across the different analyses. Instead, N varies depending on which items or variables a given analysis requires, and these sample sizes are reported alongside each result throughout the results section. Across analyses, the number of cases excluded due to missing data ranged from as few as 2 participants (1.0% of the sample) to as many as 45 participants (22.3%). The total number of cases deleted ranged from 2 (1.0%) to 45 (22.3%). Data analysis was conducted in R (version 4.5.2)^36^ via RStudio v.2025.05.1 + 513 ^37^ as well as SPSS v.31.

To answer RQ1 (i.e., whether faculty beliefs differed across the three goals of DEC), we first used the Friedman test. The Friedman test is a nonparametric alternative to repeated measures ANOVA and is used to detect differences across multiple related groups or matched samples.^38^ In our case, we compared the same faculty member’s ratings (ordinal data) of the three different goals using the Friedman test. We then followed-up with posthoc Wilcoxon-signed rank tests. The Wilcoxon signed-rank test is a nonparametric alternative to the paired-samples *t* test and is used to compare two dependent or paired samples.^39^ Since the Friedman test only indicates whether a difference exists in faculty member’s ratings of the three different goals, posthoc Wilcoxon-signed rank tests make pairwise comparisons between each pair of goals (e.g., Goal IS vs Goal FC) to determine exactly where the differences lie.

To answer RQ2 (i.e., whether faculty beliefs varied by academic rank and service role), we used Kruskal–Wallis tests and Independent-samples Mann–Whitney U tests. The Kruskal–Wallis test is a nonparametrical alternative to one-way ANOVA and is used to determine if there are statistically significant differences between three or more independent groups.^40^ This test was used to compare faculty beliefs about each goal across the three academic rank groups (Assistant, Associate, and Full Professor). The independent samples Mann–Whitney U test is a nonparametric alternative to the independent samples *t* test^41^ and is used to compare faculty beliefs about each goal between the two service roles (i.e., those with and without a graduate-education service role).

## Results and Discussion

Table 2 lists the distribution of participants based on various demographic variables, including academic rank and service role. The sample represents faculty who elected to participate in the survey and, therefore, should not be interpreted as representative of all chemistry faculty. Consequently, the findings provide insights into the beliefs of the participating faculty and are most appropriately interpreted within the context of the sample obtained.

**Table 2:** Demographics of Participants

*Variable*	*Category*	*N*
Gender Identity	Female	43
	Male	80
	Nonbinary	1
Ethnicity	White	121
	Black or African American	2
	American Indian or Alaska Native	0
	Asian	13
	Native Hawaiian or Pacific Islander	0
	Other	4
Division^a^	Analytical Chemistry	19
	Biochemistry	29
	Inorganic Chemistry	37
	Organic Chemistry	44
	Physical Chemistry	31
	Theoretical Chemistry	11
	Materials Chemistry	28
	Environmental Chemistry	6
	Medicinal Chemistry	11
	Chemical Education	24
Academic Rank	Assistant Professor	30
	Associate Professor	38
	Full Professor	94
Graduate-Related Service Role	Yes	110
	No	54

a Participants could select multiple research divisions.

Below we present results for each research question and how that relates to each of our hypotheses. We have also summarized the results in Table 3.

**Table 3:** Summary of Results

*RQ*	*Hypothesis*	*TCSR construct*	*Prediction*	*Statistical test*	*Result*	*Hypothesis Verdict*
RQ1	Hypothesis IS	Teacher’s thinking (i.e., beliefs)	Strong, near-uniform endorsement	Descriptive (% agreement)	97% agreed (86% strongly)	Supported
RQ1	Hypothesis FC	Teacher’s thinking (i.e., beliefs)	High agreement, but variable relative to other goals	Descriptive (% agreement)	96% agreed (80% strongly	Agreement high, but ranking data show FC slightly secondary to IS
RQ1	Hypothesis IR	Teacher’s thinking shaped by contextual factors (institutional incentives/prestige structures)	More variable than IS/FC	Descriptive (% agreement)	55% agreed, mostly “somewhat”	Supported
			IR distribution different from IS/FC	Friedman + posthoc Wilcoxon	χ^2^ (2) = 256.72, *p < 0.001*	Confirms differentiated pattern
RQ2	Hypothesis Academic rank	Personal factors into teacher’s thinking	Beliefs vary by academic rank	Kruskal–Wallis	No evidence of significant differences	Not supported
RQ2	Hypothesis Service role	Contextual factors into teacher’s thinking	Beliefs vary by service role	Mann–Whitney U	No evidence of significant differences	Not supported

### RQ1: Faculty Beliefs about Primary Goals of DEC

Absolute agreement: Figure 2 presents the distribution of faculty responses for each of the three goals. Two of the hypothesized goals received strong agreement. About 97% of faculty agreed strongly (86%) or somewhat (11%) that DEC aims to develop students into independent scientists (Goal IS). Similarly, approximately 96% of faculty agreed strongly (80%) or somewhat (16%) that a core goal of DEC is to prepare students to be competitive for future careers (Goal FC). In contrast, ratings for the goal of developing students who can improve their institution’s rank or reputation (Goal IR) exhibited noticeably greater variability. Approximately 55% of faculty somewhat agree (∼47%) or strongly agree (∼9%) that this represents a primary goal of DEC. The Friedman test further revealed evidence for a statistically significant difference across the three distributions χ^2^ (2) = 256.72, *p < 0.001*, n = 195. In other words, the distribution of faculty beliefs across the three goals differed. Posthoc testing through the Wilcoxon-signed rank test indicated that faculty beliefs for Goal IR were significantly different from both Goal IS and Goal FC (*p < 0.001*).

**Figure 2:**
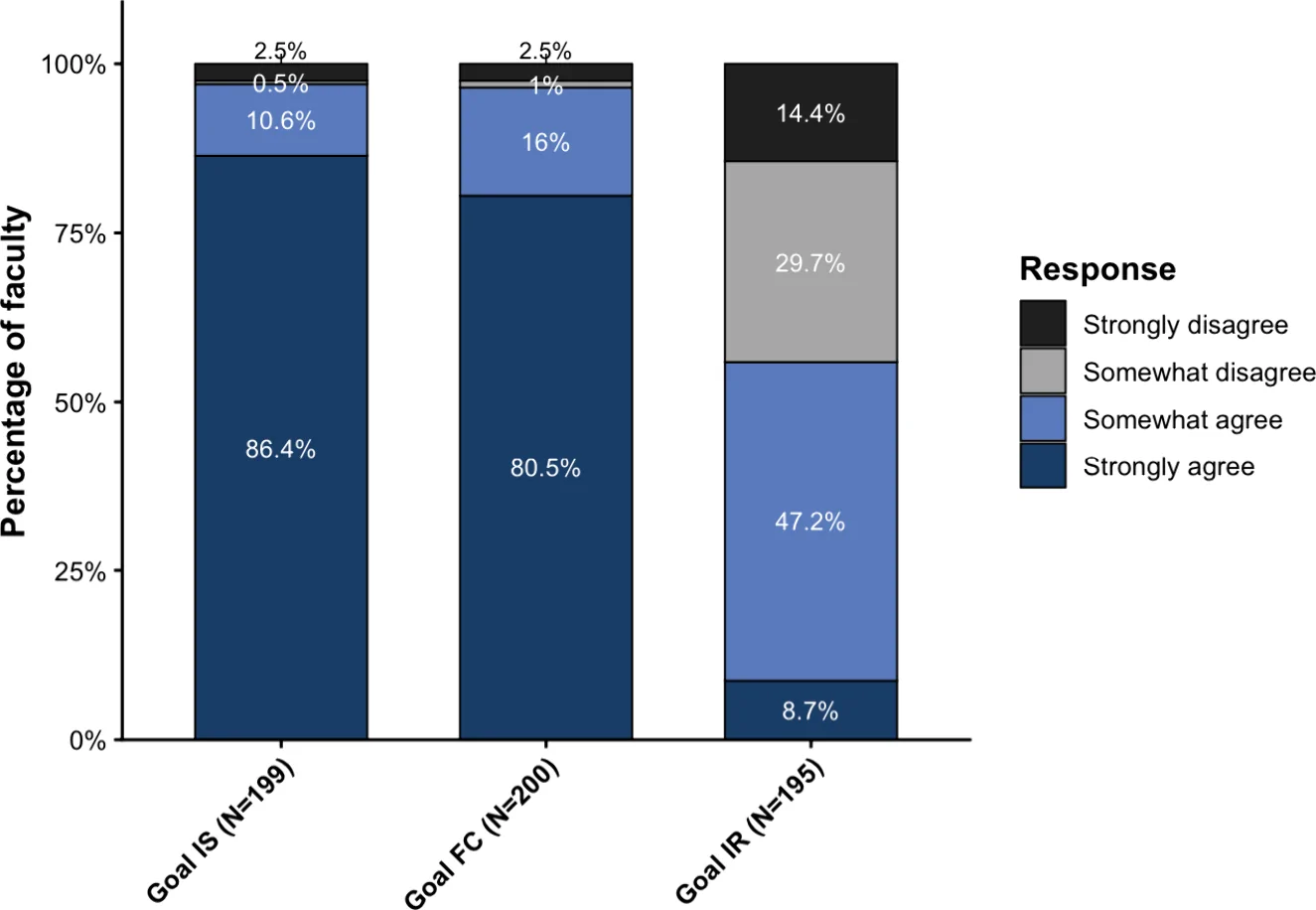
Distribution of faculty beliefs about goals of DEC.

The observed patterns of faculty agreement align with the hypothesized expectations for each doctoral education goal, although in distinct ways. Consistent with hypothesis IS, faculty demonstrated strong and near-uniform endorsement of developing independent scientists, reflecting the historical centrality of scientific independence within doctoral education in chemistry.^22–25^ In the case of preparing students for future careers (hypothesis FC), faculty also showed high overall agreement; however, this finding should be interpreted in light of the hypothesis, which anticipated variation in how career preparation is conceptualized, relative to other goals. The strong agreement observed suggests that, despite its nonvocational origins, career preparation has become broadly recognized by faculty as a primary goal of DEC. The high endorsement of Goal FC by faculty echoes the calls by NASEM^6^ and ACS.^10^ In contrast, and as predicted by the hypothesis IR, faculty beliefs regarding enhancing institutional rank or reputation were substantially more variable and significantly different from the other two goals. Notably, among faculty who endorsed Goal IR, the majority did so at the level of somewhat agree rather than strongly agree, suggesting a more reserved or conditional endorsement of the institutional rank or reputation as a doctoral education goal. This response pattern indicates that while faculty may acknowledge the relevance of institutional reputation to doctoral education, they are less inclined to fully internalize it as a guiding goal. Within the TCSR framework, this pattern suggests that contributing to institutional rank is less consistently internalized within faculty thinking as a core goal of DEC relative to student-centered goals such as career preparation and the development of scientific independence.^21^

Relative Priority: Faculty were also asked to rank the three goals to assess their relative priority. As shown in Figure 3, the ranking patterns closely mirror the absolute agreement ratings reported above. Approximately 59% of faculty ranked Goal IS as the most important goal, followed by about 46% who ranked Goal FC as their top priority. One potential explanation for the relative prioritization of Goal IS over Goal FC is that faculty may conceptualize these goals as interdependent^20^ but ordered differently, particularly when prompted to rank them. While many faculty likely view scientific independence as a key pathway through which career success is achieved, others may prioritize career readiness more explicitly as a student outcome; ranking the goals may therefore have required faculty to articulate which comes first, rather than whether both matter. At the same time, the act of ranking may have prompted faculty to reflect on enacted practices within doctoral programs, which remain heavily structured around research training, dissertation milestones, and indicators of independence (further discussed in the Implications section). In contrast, Goal IR was ranked the first priority by approximately only 7% of respondents. This indicates that even among faculty who acknowledge institutional reputation as a goal of doctoral education in chemistry, most view it as substantially less central than preparing independent scientists or supporting students’ career competitiveness.

**Figure 3:**
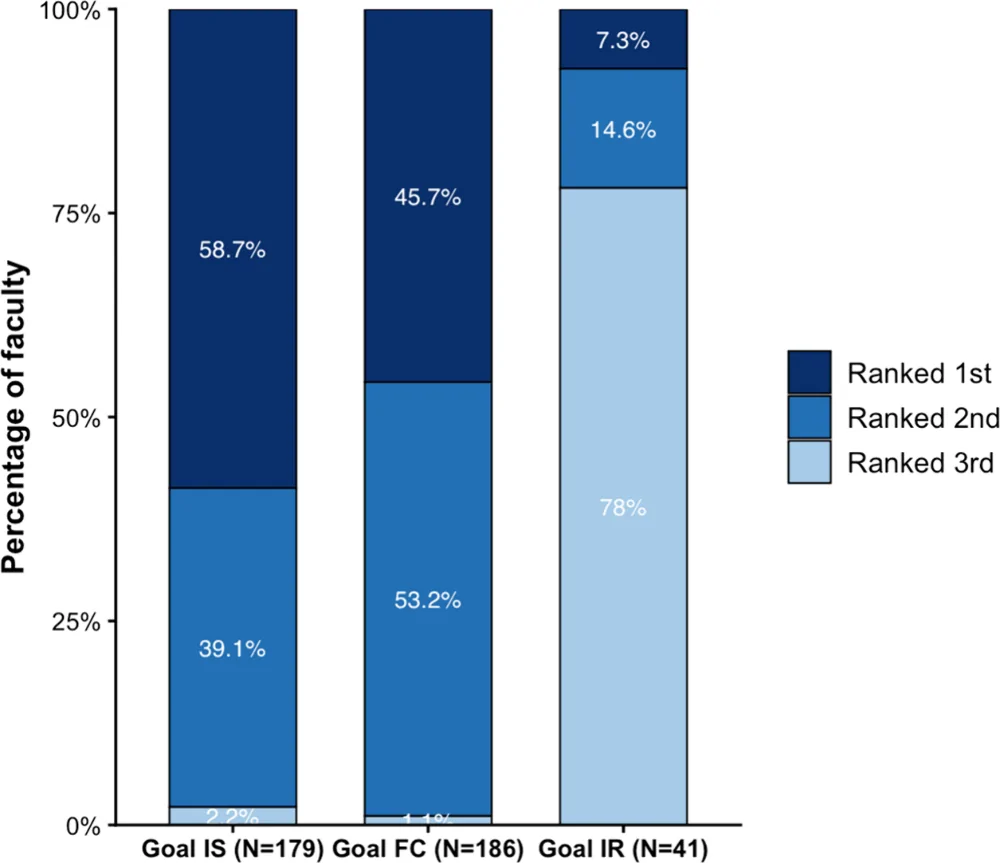
Ranking distribution of the goals.

### RQ2: Relationship of Personal and Contextual Factors with Faculty Beliefs about Goals of DEC

The TCSR model posits a bidirectional relationship between teacher thinking and personal context such that personal and contextual factors may shape faculty beliefs. To examine this relationship within the context of DEC, we collected data on two factors: (1) service role and (2) academic rank. Figure 4 and Figure 5 display the distribution for each goal based on academic rank and service role, respectively.

**Figure 4:**
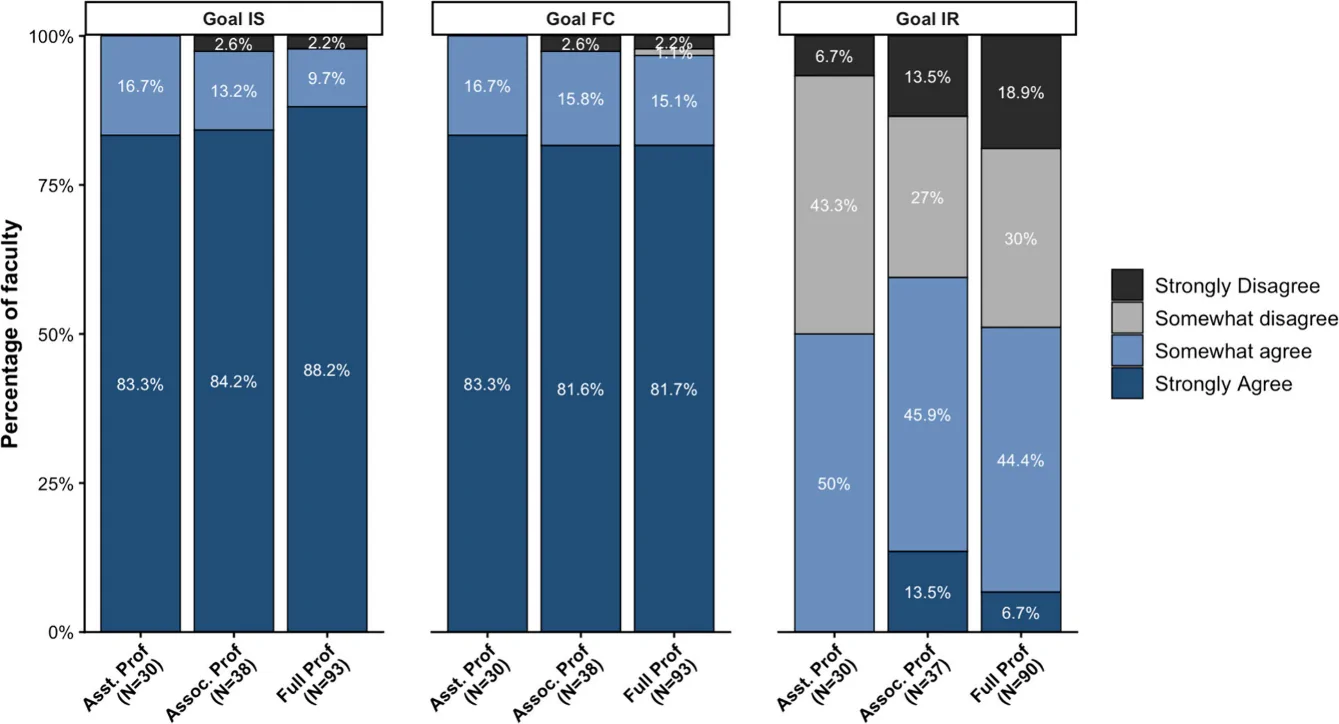
Distribution of faculty beliefs about goals of DEC based on academic rank.

**Figure 5:**
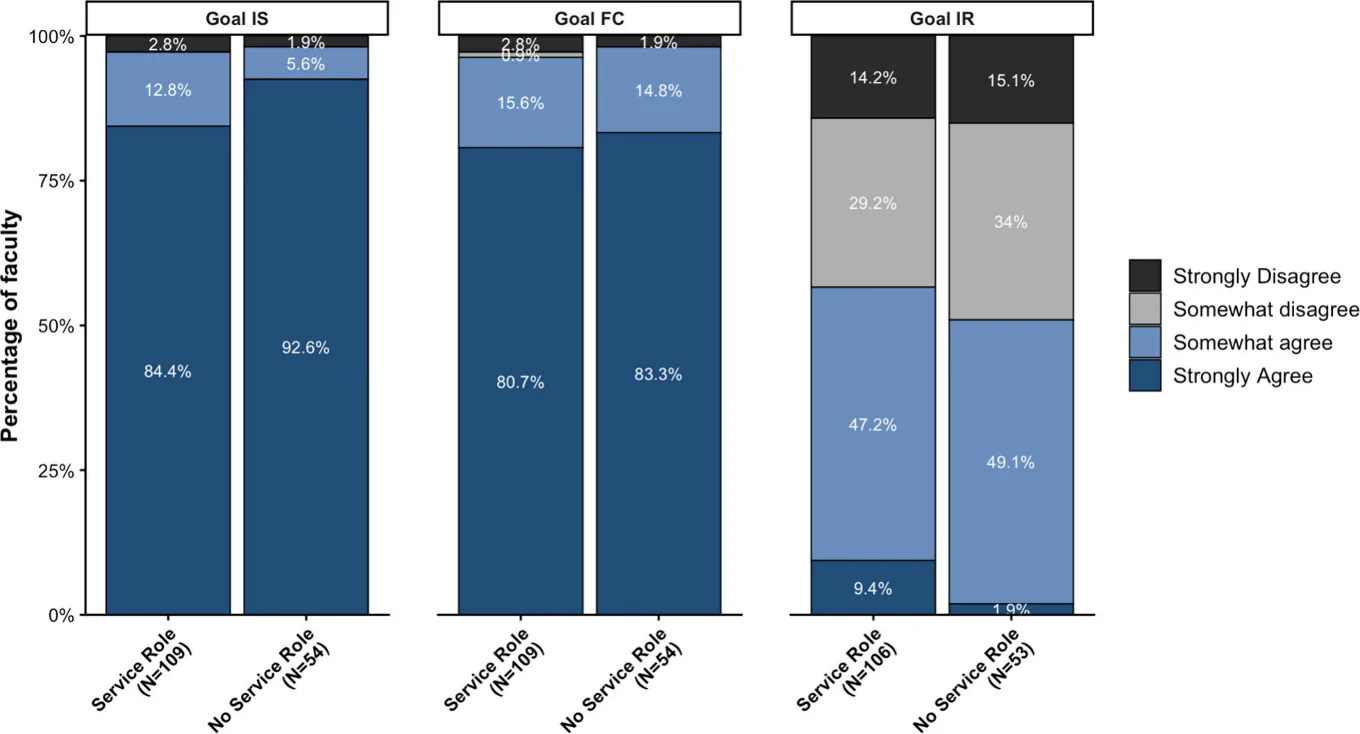
Distribution of faculty beliefs about goals of DEC based on service role.

On the basis of prior literature and the TCSR framework, we hypothesized that faculty beliefs about the goals of DEC might vary by academic rank and participation in doctoral-education service roles, given differences in professional socialization and exposure to programmatic and institutional contexts. However, for all three goals, results indicated no evidence for statistically significant differences by academic rank with negligible effect sizes. Similarly, across all goals, there was no evidence of statistically significant differences between faculty with doctoral-education service roles and those without, and effect sizes ranged from negligible to very small (see Supporting Information for statistical details).

No evidence for a statistically significant difference based on academic rank and service role in the current study suggests a high degree of shared belief structures regarding the goals of DEC. Research on doctoral education suggests that disciplinary norms and shared cultural expectations play a dominant role in shaping faculty conceptions of doctoral training, often outweighing differences associated with career stage or administrative responsibility.^30,42^ In chemistry, the relative standardization of doctoral program structures may contribute to convergence in beliefs about overarching goals, even among faculty who occupy different roles or ranks. From a TCSR perspective, alignment in faculty thinking represents a critical precondition for change, as beliefs serve as the lens through which new structures and practices are interpreted and enacted.^21^ Thus, the observed homogeneity in beliefs about DEC goals may facilitate reform by enabling departments to focus less on negotiating what doctoral education should achieve and more on aligning programmatic elements and practices to more effectively realize these shared goals.

## Implications

Together, the results show strong faculty consensus around student-centered goals of scientific independence and career preparation, alongside more variable endorsement of an institution-focused goal related to rank or reputation. These patterns give rise to two broad sets of implications: first, for understanding the tension between shared faculty goals and the research-centered structure of chemistry doctoral programs and, second, for identifying promising directions for future research that examine how additional contextual factors shape faculty beliefs about doctoral education.

### Implication 1

The present findings reveal an important tension in DEC: faculty overwhelmingly agree that developing independent scientists as well as preparing students to be competitive for future careers are core goals of DEC, yet the prevailing structure of DEC remains primarily oriented toward achieving scientific independence through intensive research training. The emphasis on intensive research training is consistent with the historical conception of the PhD as a research degree.^22–25^ One common notion is that achieving the goal of developing independent scientists is assumed to simultaneously prepare them for their careers.^20^ However, national data consistently show that a substantial proportion of chemistry PhD graduates pursue careers that are not primarily research-intensive in a variety of sectors.^3,8^ CPT’s 2015 survey of PhD chemistry recipients also reported substantial disagreement among graduates regarding how well their doctoral education prepared them for careers outside the research-intensive academic pathway^8^ casting doubt on the assumed relationship between the two primary goals. Additionally, the goal of developing independent scientists is centered on students, but if departments set this goal primarily so to achieve the needs of the institution and research enterprises,^6,43,44^ it can be questioned if this truly represents a student-centered goal. Taken together, these results suggest that although faculty broadly endorse both Goal IS and Goal FC strongly, existing programmatic structures may be insufficiently aligned to support both goals in practice. This tension also parallel themes that have been raised in ongoing national discussions regarding the future of graduate education and scientific workforce development.^45^

Importantly, the strong consensus around both goals represents an opportunity rather than an obstacle for reform. Rather than assuming that existing structures uniformly support all desired outcomes, reform efforts may benefit from structured discussions in which faculty explicitly map whether and how each element attends to the goals of developing independent scientists, as well as preparing students for diverse careers. Such reflective alignment work can help make implicit assumptions visible, identify gaps between goals and enactment, and create a shared basis for evaluating potential modifications to the programmatic elements of DEC. In this way, faculty dialogue focused on programmatic coherence may serve as a critical mechanism for translating consensus on goals into meaningful and sustainable reforms.

Departmental seminars provide one illustrative example of how such an alignment work might unfold in practice. In many chemistry doctoral programs, seminars are traditionally structured around research talks by faculty, visiting scholars, and advanced graduate students and thus primarily function as exposure to cutting-edge research. When mapped against faculty-endorsed goals, this structure clearly supports goal IS by modeling how independent scientists frame research problems, justify methodological choices, and communicate findings to disciplinary audiences. At the same time, this mapping exercise can reveal more limited alignment with goal FC, prompting consideration of how seminars might also attend to career competitiveness. For instance, periodically featuring speakers from teaching-focused institutions, industry, government laboratories, policy offices, scientific publishing, or outreach organizations has the potential to expose doctoral students to the expectations, norms, and skill sets associated with careers beyond research-intensive academia. Such seminars can make explicit how disciplinary expertise is applied in nonacademic contexts and surface competencies valued in these roles, including communication with nonspecialist audiences, teamwork, project management, regulatory awareness, and pedagogical expertise. Additionally, speakers from these sectors may provide concrete insights into hiring processes, career trajectories, and professional evaluation criteria. Viewed collectively, this example illustrates how modest, intentional adjustments to an existing programmatic element identified through faculty-led alignment discussions may allow departments to better support both of these goals.

### Implication 2

The variability observed in responses to Goal 3 points to the importance of examining additional contextual factors that may condition how faculty interpret this goal. We found no evidence for statistical difference in faculty beliefs about goal 3 based on service role. However, prior higher education research suggests that faculty beliefs and behaviors are shaped not only by individual characteristics but also by the organizational environments in which they work. Faculty members interpret academic priorities through institutional missions, reward structures, and expectations regarding research, teaching, and service.^30,31,46,47^ For example, institutions differ substantially in the extent to which research productivity, grant acquisition, doctoral production, and national visibility are emphasized within faculty evaluation and departmental success metrics.^48,49^ Such differences may shape the salience of institution-focused goals. Faculty at institutions classified as “Very High Research Spending and Doctorate Production” (R1) may operate within environments where publication output, external funding, and institutional reputation are more tightly coupled to departmental evaluation than at “High Research Spending and Doctorate Production” (R2) institutions. Research on academic prestige and stratification further suggests that highly research-intensive institutions often participate in organizational systems that place greater emphasis on reputation, rankings, and visibility as markers of success.^29^ Future research that explicitly examines how institutional context and enacted practices interact to shape faculty beliefs about Goal 3 would provide valuable insight into when and why prestige-oriented goals gain traction in DEC.

### Implication 3

Although this study was conducted within the context of U.S. chemistry doctoral education, the patterns observed here resonate with broader international transformations in doctoral education. Across many countries, doctoral programs have historically been organized around the preparation of independent researchers through apprenticeship-based research training, yet they increasingly face expectations to prepare graduates for diverse careers, contribute to innovation systems, and address societal needs.^50,51^ In Europe, for example, doctoral education reforms have emphasized researcher development, transferable skills, employability, and societal relevance alongside traditional research training.^52,53^ Faculty consensus observed here by US-based faculty around scientific independence and career preparation may reflect a challenge that extends beyond chemistry and beyond the United States: how doctoral programs can reconcile long-standing conceptions of the PhD with evolving expectations from students, employers, funding agencies, and society. The variability surrounding the institution-focused goal further suggests that tensions between educational missions and prestige-driven incentive structures may be a common feature of contemporary doctoral education systems. Comparative studies across countries and disciplines would therefore provide valuable insights into whether the patterns observed here reflect broader international trends in faculty conceptions of doctoral education.

## Conclusions

This study provides evidence from a national sample of chemistry faculty beliefs about the primary goals of doctoral education, offering a broad empirical perspective on how doctoral education is conceptualized across the discipline. Faculty demonstrated strong consensus around student-centered goals (developing independent scientists and preparing students for future careers) while exhibiting more variable and less strongly prioritized endorsement of institution-focused goal (improving institution’s rank or reputation). Importantly, this consensus held across personal and contextual factors examined in the study, suggesting that faculty beliefs about doctoral education goals are widely shared among research-active chemistry faculty, one of the significant stakeholders of DEC. By clarifying where faculty consensus is strongest and where beliefs are more diffuse, this work establishes a foundation for reform efforts focused on aligning programmatic structures with faculty-endorsed goals. More broadly, the results underscore the value of grounding doctoral education reform in empirically documented faculty beliefs as a means of supporting coherent, sustainable change in chemistry doctoral education.

## Limitations

Several limitations should be considered when interpreting the results of this study.

First, this study relies on faculty’s self-reported beliefs about the goals of doctoral education in chemistry. As such, the findings reflect how faculty conceptualize and articulate the purposes of doctoral training rather than providing direct evidence of institutional priorities or program-level implementation. This distinction is important when interpreting the results, which are intended to characterize faculty thinking rather than to evaluate doctoral education outcomes.

Second, although this study examined selected personal and contextual factors such as academic rank and doctoral education service roles, other potentially influential contextual variables were not included. Institutional characteristics such as research intensity, funding models, disciplinary subfields, and local reward structures may further condition faculty beliefs and merit additional investigations. In addition, participation in the survey was voluntary, raising the possibility of self-selection bias; faculty with a stronger interest in doctoral education may be overrepresented.

Finally, the cross-sectional nature of the survey limits the analysis to beliefs at a single point in time. Faculty beliefs may evolve in response to changes in the institutional context, workforce conditions, or professional experiences. Longitudinal approaches would therefore be valuable for examining how faculty beliefs about doctoral education goals develop over time and how they interact with enacted programmatic practices.

## Supplementary Material





## Notes

Participants’ responses to “other goals” item, Kruskal–Wallis test results for goals and academic rank, and Mann–Whitney U test results for goals and service role (PDF), (DOCX)
