# Supplementary material for: Faculty Beliefs about the Goals of Doctoral Education in Chemistry: Results from a National Survey
Source: J Chem Educ. 2026 Oct 13;103(11):6394–404. doi: 10.1021/acs.jchemed.6c01087 (PMC13757759; doi:10.1021/acs.jchemed.6c01087)
Supplement: Supplementary file 1 [file ed6c01087_si_001.pdf]

Faculty Beliefs About the Goals of Doctoral Education in Chemistry: Results from a National Survey

Pallavi Nayyar[1], Benedicta Donkor[2], Jordan Harshman[1]*

1. Department of Chemistry, University of Iowa, Iowa City, Iowa, United States, 52246

2. Department of Chemistry, Middle Tennessee State University, Murfreesboro, Tennessee United States, 37132

PARTICIPANTS' RESPONSES TO "OTHER GOALS" ITEM

Faculty were invited to indicate whether they believed there were additional primary goals of doctoral education beyond those listed in the survey (Figure S1).

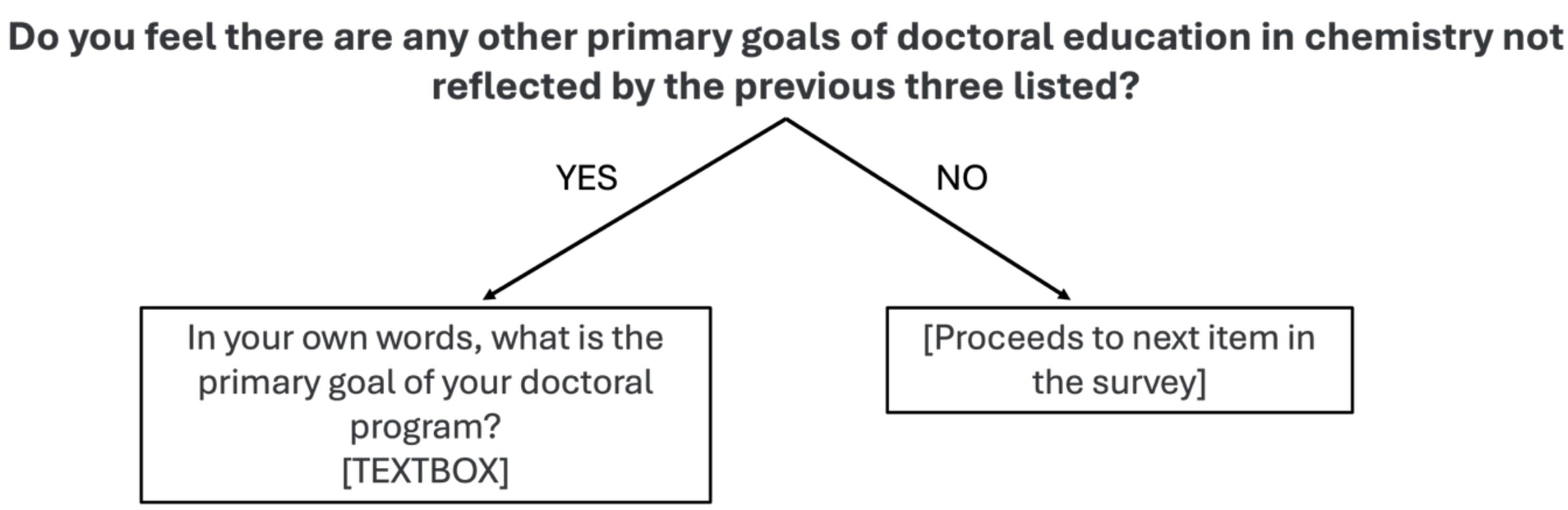

Figure S1. Survey items related to "other" goals of doctoral education.

Approximately 67% of participants (n = 116) indicated that there were no additional goals of doctoral education beyond those listed, while 33% (n = 58) responded affirmatively. Among the 58 faculty who indicated the presence of additional goals, 55 provided a free-text description, of which 49 responses were retained for analysis; six responses were excluded because they did not address the prompt. The retained responses were coded using the framework developed by Donkor et al. (2024)[1], supplemented with a categorization based on 21st-century skills[2] (Table S1). Notably, the high proportion of respondents indicating no additional goals lends further confidence to the completeness of the primary goal framework used in this study. Among the remaining responses, most aligned with existing primary goals or emphasized

skill-based outcomes commonly associated with specific programmatic elements of doctoral education, including competencies such as expertise, critical thinking, communication, leadership, and teaching typically cultivated through coursework, research training, teaching assistantships, or seminars.

Responses coded as Goal 1/2/3: Approximately 20% of responses (10 total) were categorized under the existing goals of developing independent scientists, preparing students for future careers, or contributing to institutional rank or reputation. Although these faculty selected "yes" when asked whether additional goals exist, their written responses aligned with goal categories already defined in the Donkor et al. (2024)[1] codebook. For example, one faculty member wrote, *"The only goal any program has is to increase their rankings through papers funding and citations."* This response directly reflects the goal of institutional rank or reputation.

Responses coded as secondary goals identified by Donkor et al. 2024: An additional 30% of responses (15 total) aligned with secondary goals previously identified by Donkor *et al.* 2024[1], including disciplinary expertise, critical or independent thinking, and the generation of novel research. Faculty articulated these goals using statements such as *"Becoming content-area experts in a subfield"* [expertise], *"Developing thinkers and learners"* [critical/independent thinkers], and *"To advance the chemistry discipline in terms of our understanding of chemistry"* [novel research].

Responses coded as 21st Century skills: Approximately 39% of responses (19 total) referenced one or more 21st-century skills[2]. These included learning skills (e.g., creativity, collaboration, communication), literacy skills (e.g., information, media, or technology literacy), and life skills (e.g., leadership, productivity, social skills). Representative responses coded in this category include *"communicating technical ideas and their impacts to various audiences"* [learning skills], *"Science and data literacy and science communication"* [learning + literacy skills], and *"In addition to those goals mentioned above we also want students to learn how to teach, train and manage other people in a laboratory setting."* [life skills].

Responses coded as goal and 21st Century skills: The remaining 10% of responses (5 total) articulated combinations of previously identified goals and 21st-century skills. For instance, one faculty member wrote, *"learn to carry out research, think critically about the purpose, methodology and report results in a professional manner."* [critical/independent thinkers + learning skills]. Another noted, *"Develop students who can identify important socio-scientific problems for research focus meaning they understand that there is bias in science in terms of the science we pursue the value of that science and who it impacts. We need to develop students who can effectively communicate about their research to the public."* [independent scientist + learning skills].

Table S1. Coding of open-ended responses to "In your own words, what is the primary goal of your doctoral program?"

No. of Participants	*Goals mentioned*	
**9**	**Life skills**	
**4**	**Life skills**	**Learning skills**
**1**	**Life skills**	**Expertise**
**5**	**Learning skills**	
**1**	**Learning skills**	**Novel research**
**1**	**Learning skills**	**Literacy skills**
**1**	**Learning skills**	**Independent scientist**

**4**	**Expertise**		
**5**	**Independent scientist**		
**1**	**Independent scientist**	**Novel research**	
**1**	**Independent scientist**	**Diverse careers**	
**1**	**Independent scientist**	**Learning skills**	**Diverse careers**
**4**	**Critical/Independent learner/thinker**		
**1**	**Critical/Independent learner/thinker**	**Learning skills**	
**2**	**Institution Rank**		
**2**	**Diverse careers**		
**6**	**Novel research**		

KRUSKAL-WALLIS TEST RESULTS FOR GOALS AND ACADEMIC RANK

Diverse Careers: A Kruskal-Wallis test indicated no evidence of a significant difference among faculty beliefs about Goal 1 based on academic rank/title: H(2) = .077, $p = .962$ $\eta^2 = .0005$, *indicating a negligible effect.* Median: 4 (Assistant Professor, n= 30); 4 (Associate Professor, n= 38); 4 (Full Professor, n= 93)

Independent Scientists: A Kruskal-Wallis test indicated no evidence of a significant difference among faculty beliefs about Goal 2 based on academic rank/title $H(2) = 0.58$, $p = .75$, $\eta^2 = .004$,

indicating a negligible effect. Median: 4 (Assistant Professor, n= 30); Median: 4 (Associate Professor, n= 38); 4 (Full Professor, n= 93)

Institutional Rank: A Kruskal-Wallis test indicated no evidence of a significant difference among faculty beliefs about Goal 3 based on academic rank/title: $H(2) = 1.48$, $p = .48$, $\eta^2 = .010$, suggesting a negligible effect. Median: 2.5 (Assistant Professor, n= 30); 3 (Associate Professor, n= 37); 3 (Full Professor, n= 90)

MANN-WHITNEY U TEST RESULTS FOR GOALS AND SERVICE ROLE

Diverse Careers: A Mann–Whitney U test indicated no evidence of a significant difference in faculty beliefs about Goal 1 based on whether they hold a service role in graduate studies (n = 54) or no service role (n = 109), U=2859.5, Z=−0.437Z, $p = .662$. Effect sizes were trivial (r=.03; Vargha–Delaney $A_{\text{No service>Service}}$=0.514; Cliff's $\delta_{\text{No service−Service}}$=0.03) Medians were equal (4 vs 4).

Independent Scientists: A Mann–Whitney U test indicated no evidence of a significant difference in faculty beliefs about Goal 1 based on whether they hold a service role in graduate studies (n = 54) or no service role (n = 109), U=2704.5, Z=−1.447, $p = .148$. Effect sizes were small (r=.11; Vargha–Delaney $A_{\text{No service>Service}}$=0.541; Cliff's $\delta_{\text{No service−Service}}$=0.08) Medians were equal (4 vs 4).

Institutional Rank: A Mann–Whitney U test indicated no evidence of a significant in faculty beliefs about Goal 1 based on whether they hold a service role in graduate studies (n = 53) or no service role (n = 106), U=3062, Z=0.998, $p = .318$. Effect sizes were small (r=.08; Vargha–Delaney $A_{\text{No service>Service}}$=0.545; Cliff's $\delta_{\text{No service−Service}}$=0.09) Medians were equal (3 vs 3).

REFERENCES

(1) Donkor, B.; Collini, M. A.; Harshman, J. *The Goal of Doctoral Education in Chemistry: Faculty Perspectives | Journal of Chemical Education*. https://pubs.acs.org/doi/full/10.1021/acs.jchemed.4c00087 (accessed 2026-05-08).

(2) Council, N. R.; Education, D. of B. and S. S. and; Education, B. on S.; Assessment, B. on T. and; Skills, C. on D. D. L. and 21st C. *Education for Life and Work: Developing Transferable Knowledge and Skills in the 21st Century*; National Academies Press, 2012.
